# Molecular Divergence and Species Delimitation of the Cultivated Oyster Mushrooms: Integration of IGS1 and ITS

**DOI:** 10.1155/2014/793414

**Published:** 2014-01-21

**Authors:** Farhat Ahmadi Avin, Subha Bhassu, Yee Shin Tan, Pedram Shahbazi, Sabaratnam Vikineswary

**Affiliations:** ^1^Mushroom Research Centre (MRC), University of Malaya, 50603 Kuala Lumpur, Malaysia; ^2^Division of Biotechnology, Institute of Biological Sciences, Faculty of Science, University of Malaya, 50603 Kuala Lumpur, Malaysia; ^3^Division of Genetics and Molecular Biology, Institute of Biological Sciences, Faculty of Science, University of Malaya, 50603 Kuala Lumpur, Malaysia

## Abstract

Identification of edible mushrooms particularly *Pleurotus* genus has been restricted due to various obstacles. The present study attempted to use the combination of two variable regions of IGS1 and ITS for classifying the economically cultivated *Pleurotus* species. Integration of the two regions proved a high ability that not only could clearly distinguish the species but also served sufficient intraspecies variation. Phylogenetic tree (IGS1 + ITS) showed seven distinct clades, each clade belonging to a separate species group. Moreover, the species differentiation was tested by AMOVA and the results were reconfirmed by presenting appropriate amounts of divergence (91.82% among and 8.18% within the species). In spite of achieving a proper classification of species by combination of IGS1 and ITS sequences, the phylogenetic tree showed the misclassification of the species of *P. nebrodensis* and *P. eryngii* var. *ferulae* with other strains of *P. eryngii*. However, the constructed median joining (MJ) network could not only differentiate between these species but also offer a profound perception of the species' evolutionary process. Eventually, due to the sufficient variation among and within species, distinct sequences, simple amplification, and location between ideal conserved ribosomal genes, the integration of IGS1 and ITS sequences is recommended as a desirable DNA barcode.

## 1. Introduction

*Pleurotus* genera (Pleurotaceae, Agaricales, and Basidiomycetes) also known as oyster mushrooms are worldwide distributed macrofungi [1] that comprise various kinds of highly priced edible mushrooms [2]. Edible mushrooms are also a complete source of nutrition. They are known for their high levels of fibers, carbohydrates, amino acids, proteins, vitamins, and many other minerals [3].

To date, identification of edible mushrooms particularly *Pleurotus* genus has been constrained due to various factors. Cultivated edible mushrooms that belong to the *Pleurotus* genus have been principally discriminated by their morphological features such as shape, colour and size of hymenophore, length, thickness and colour of stipe, yield, and duration for maturation [4]. However, the notion of species differentiation based on environmental factors can be unreliable and lead to misidentification as well as erroneous taxonomic conclusions [5]. Moreover, the number of fungal species that can be classified easily by morphological factors is not considerable [6].

Even in a country like Malaysia which has a vast diversity of mushrooms and where the medicinal and dietary properties of various edible fungi are widely known [7], not many studies have been conducted that concentrate on the phylogenetic relationships and discrimination of cultivated edible fungi, particularly by using molecular methods.

The advantages of identification are obvious. All cultivated varieties of fungi can face loss of genetic diversity and inbreeding effects [8] that force farmers to introduce new hybrids in their farms. The biggest concern would be of adaptation of new lines to the new environment and of tracking lines that remain stable in various environmental parameters. Classification of mushroom species and genus through the introduction of cultivated line transfers across farms and also between Asian countries could also be a challenge as the demand of this genus is rapidly expanding.

Moreover, classification of fungal species using DNA barcodes is a reliable and effective tool and it can be implemented at any period of hyphal development. With the appearance of DNA based molecular methods, several molecular techniques, including SSR [9], RAPD [10], AFLP [8], and the sequences of mitochondrial SSU rRNA [11], cytochrome oxidase genes [12, 13], and partial EF1*α* and RPB2 gene [14] have been used to validate mushroom species. The existing investigations have no doubt assisted in fungal identification; however, some of the reports could not accurately categorize the fungi genera, particularly intraspecies of *Pleurotus*.

Up to the present, numerous studies demonstrated that internal transcribed spacer 1 and 2 regions [15] possessed a great potential to distinguish between species of fungi [16–18]. According to Schoch et al. [18], ITS region has the highest probability of successful identification for the broadest range of fungi, with the most clearly defined divergence level between inter- and intraspecific variation. However, a number of previously reported studies demonstrated that ITS is not a powerful tool to distinguish many closely related fungal species [19, 20] as well as intragenus [5, 6]. Although there are thoughts in inefficiency of this marker, Schoch and Seifert [21] still believe that ITS as a DNA barcode provides the best possible path to achieve this goal in fungi.

The intergenic spacer (IGS) regions are two noncoding and highly variable DNA units [22] that are located between conserved sequences of 25S, 5S, and 18S within the nuclear rRNA gene [23]. IGS1 is normally shorter than IGS2 and can be easily used for discriminating strains of the uncultured species [24]. However, only a limited number of studies have focused on IGS region as a systematic tool for species delimitation of the commonly cultivated edible mushrooms.

According to Dupuis et al. [25], it has been shown that all marker groups have relatively equal success in delineating closely related species and that using more markers increases average delimitation success. Hence, this study aimed (i) to develop new pairs of primer for amplification and sequencing of two highly variable regions of intergenic spacer (IGS) 1 and internal transcribed spacer (ITS) 1 and 2, (ii) to investigate the molecular phylogeny and taxonomic relationships of common cultivated species of *Pleurotus* using variation at the IGS1 and ITS regions of nuclear ribosomal DNA, and (iii) to assess the ability of combined IGS1 and ITS regions as DNA barcode.

## 2. Materials and Methods

### 2.1. Fungi and Culture Preparation

The experimental fungal samples were mainly collected from several Malaysian mushroom farms and some obtained from the Fungal Biotechnology Laboratory, Institute of Biological Sciences, Faculty of Science, University of Malaya. Strains and species used in this study (Table 1) were morphologically identified using taxonomic keys by the Mushroom Research Centre (MRC), University of Malaya. Axenic dikaryon cultures were prepared by transferring a piece of tissue from the context of the fruit body to a potato dextrose agar (PDA) plate. The Petri dishes were incubated at 28 ± 2°C for 14 days.

### 2.2. DNA Extraction

Mycelia were directly scraped (50 mg) from the surface of the solid medium. Sterilized quartz (50 mg) and sufficient 2% SDS buffer were added to the samples [26]. The samples were then incubated for 45 minutes at 58°C. After homogenization and cell lysis steps, the tubes were centrifuged at 16000 ×g for 10 minutes. Next, the samples were extracted twice using CHCl_3_ : isoamyl alcohol (24 : 1). After precipitation, the DNA pellet was dissolved in 50 *μ*L of TE buffer and maintained in −20°C for further analysis.

### 2.3. Primer Design

**Figure 1:**
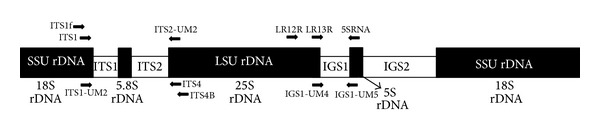
Primer binding sites used to amplify IGS1 and ITS regions. The arrowheads show the 3′ end of each primer. LSU rDNA represents the large subunit ribosomal DNA and LSU rDNA represents the small subunit ribosomal DNA.

Two previously reported primers of LR12R and LR13R (as forward) and one primer of 5SRNA (as reverse) [27, 28] were employed to recover the nucleotide sequence of partial large subunit rRNA (25S), complete intergenic spacer 1 (IGS1), and partial 5SrRNA (Table 2). Moreover, four previously published primers of ITS1 and ITS1f (as forward), and ITS4 and ITS4B (as reverse) [15, 29] were used to recover the nucleotide sequences of partial 18S, complete internal transcribed spacer (ITS) 1, 5.8S, internal transcribed spacer (ITS) 2, and partial 28S (Table 2). Briefly, the obtained sequences were aligned using MEGA4.0 software [30], and the conserved regions were detected by DNAsp v. 5.0 [31]. The new pairs of primer were designed with NCBI Primer-BLAST [32, 33]. The structure of the ribosomal RNA in *Pleurotus* genus and the orientation and location of the used PCR primers, including published and unpublished, are schematically shown in Figure 1.

### 2.4. PCR Amplification Protocol

The amplification of IGS1 region was performed in a reaction volume of 50 *μ*L using a thermocycler. The reaction mixture contained 100 ng (0.5 *μ*L) of template DNA, 2.0 mM MgCl_2_, 0.3 *μ*M of each primer, 0.25 mM of each dNTPs, adequate amount of 5x *Taq* buffer, and 2 units of Go*Taq* Flexi DNA Polymerase (Promega). The cycling parameters were initial denaturation at 95°C for 3 minutes; followed by 28 cycles including denaturation at 94°C, annealing at 54°C, and extension at 72°C for 40 seconds; and finalized by extension at 72°C for 10 minutes. In order to observe the amplicons by gel documentation system, PCR products were loaded in 1.2% agarose gel, followed by electrophoresis separation, and then stained with ethidium bromide (EtBr).

### 2.5. Purification of PCR Product and Alignment

NucleoSpin Extract II Kit (Chemopharm) was used to purify the PCR products. Amplicons were sequenced in both reverse and forward directions by an ABI 3730XL automated sequencer [34]. Revision of chromatograms was performed by Chromas Lite v. 2.01 program (http://technelysium.com.au/?page_id=13). Sequences were then aligned using MEGA 4.0 software [30]. Using the new BankIt submission tool, depositing of sequences in the GenBank (National Centre for Biotechnology Information (NCBI)) was carried out (the accession numbers are shown in Table 1). Through a BLAST search, eight highly homologous sequences of IGS1 and seven of ITS regions were retrieved from GenBank as references material (Table 3).

### 2.6. Phylogenetic and Statistical Analyses

The unweighted pair group method with arithmetic mean (UPGMA) method was used to construct the phylogenetic trees. Bootstrap phylogeny analysis was done with 1000 replications to statistically test the trees. The pairwise genetic distances were calculated by maximum composite likelihood method using MEGA 4.0 program [30]. DNAsp v. 5.10.00 software was employed to compute haplotype data file [31]. To estimate the significance of variance within and among the species and to quantify the extension of families and species differences, the AMOVA (analysis of molecular variance) was calculated by Alrequin v. 3.50 program [35]. The Network ver. 4.6.0.1 software (http://www.fluxus-engineering.com/) was implemented to estimate phylogenetic relationships among the unique haplotypes. This was achieved by constructing a genealogical network tree and the median joining (MJ) algorithm [36].

## 3. Results

At first, new pairs of primer for amplification and sequencing of two highly variable regions of intergenic spacer (IGS) 1 and internal transcribed spacer (ITS) 1 and 2 were designed (Table 2). The potential of integration of IGS1 and ITS regions as inter- and intraspecies marker was examined. The efficiency of three conserved regions including IGS1, ITS, and a combination of IGS1 and ITS sequences was compared. Several parameters were individually computed for each region (Table 4). Three conserved regions were separately detected for each region (1: 126–227, 2: 355–400, and 3: 430–491 (IGS1) and 1: 1–111, 2: 363–466, and 3: 623–702 (ITS 1 and 2)).

Secondly, by use of the same samples, the IGS1 and ITS regions gave rise to 14 and 12 unique haplotypes, respectively. However with the integration of IGS1 and ITS, the number of haplotypes rose to 16, which demonstrated that the combination of IGS1 and ITS regions yielded higher intraspecies variation (Table 4). Thus this integration would be a good benchmark for resolving the molecular phylogeny and taxonomic relationships of common cultivated species of *Pleurotus*. The potential of these regions was further tested using various statistical tests. Analysis of molecular variance (AMOVA) of IGS1 sequences revealed lower percentage of variation within the samples compared to among the samples (among: 88.98%; within: 11.02%). However, AMOVA of ITS sequences showed lower variation within the species (7.27%) and high divergence among the species (92.73%). In contrast, the combination of IGS1 and ITS regions fulfilled the prerequisite of being a potential marker by demonstrating 91.82% of variation among and 8.18% within the sequences (Table 4).

Thirdly, to recommend an efficient sequence-based DNA barcode for *Pleurotus* genus, the combined IGS1 and ITS regions were used. The potential of this integrated sequence was then demonstrated by various statistical analyses. Population pairwise FST (spatial genetic differentiation) *P* values based on IGS1 sequences were calculated for eight groups of species including *P. cystidiosus* (1), *P. eryngii* (2), *P. nebrodensis* (3), *P. ostreatus* (4), *P. pulmonarius* (5), *P. floridanus* (6), *P. sapidus* (7), and *P. citrinopileatus* (8). There was no significant difference among *P. floridanus*, *P. citrinopileatus* and *P. sapidus* as well as *P. nebrodensis*, *P. eryngii* var. *ferulae* and *P. eryngii*. Moreover, these results were confirmed by the exact test of sample nondifferentiation based on haplotype frequencies (*P* ≤ 0.05). In contrast, the significant differences among the groups of *P. floridanus*, *P. citrinopileatus*, and *P. sapidus* were obtained by integration of IGS1 and ITS regions. In addition, higher FST and differentiation *P* values among *P. nebrodensis*, *P. eryngii* var. *ferulae*, and *P. eryngii* were observed.

**Figure 2:**
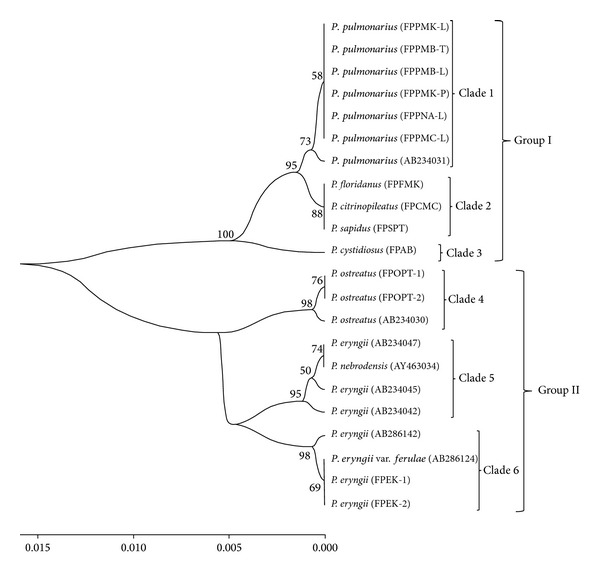
Phylogenetic tree constructed based on IGS1 sequences of experimental samples using UPGMA method. The sequences of AB234031 (*P. pulmonarius*); AB234030 (*P. ostreatus*); AB234045, AB234042, AB286142, and AB234047 (*P. eryngii*); AY463034 (*P. nebrodensis*); and AB286124 (*P. eryngii* var. *ferulae*) were downloaded from NCBI GenBank. Numbers close to branches indicate 1000 replication of bootstrap test and the codes refer to sample ID.

Homology search analysis by NCBI BLAST was performed for IGS1 sequences and eight published sequences which had great similarity with the experimental strains which were detected. The most homologous sequences taken from the BLAST search were included in the current study as references, *P. pulmonarius* (AB234031-Japan); *P. ostreatus* (AB234030-Japan); *P. eryngii* (AB234047-Japan); *P. nebrodensis* (AY463034-China); *P. eryngii* (AB234045-Japan); *P. eryngii* (AB234042-Japan); *P. eryngii* (AB286142-Japan); *P. eryngii* var. *ferulae* (AB286124-Japan). However, through our BLAST search, the reference sequences were only found for the species of *P. eryngii*, *P. pulmonarius*, and *P. ostreatus* and there was no record for the sequences of *P. sapidus*, *P. floridanus*, *P. cystidiosus*, and *P. citrinopileatus*, meaning that the sequences obtained in this study had been deposited to NCBI GenBank for the first time. The phylogenetic tree was constructed based on the sequences of IGS1 from our study (Table 1) in addition to the above-mentioned GenBank material (Table 3, Figure 2).

In addition to the IGS1 sequences, BLAST search analysis was performed for ITS sequences by NCBI BLAST. Eight sequences which had great similarity were downloaded from NCBI GenBank (HM561973 (*P. ostreatus*), AB115050 (*P. pulmonarius*), AB286159 (*P. eryngii* var. *ferulae*), AB286173 and AB286174 (*P. eryngii*), EU424308 (*P. nebrodensis*), and AY315805 (*P. cystidiosus*)).

The obtained sequences of IGS1 and ITS regions in addition to those previously deposited into GenBank were employed to construct a combined phylogenetic tree. The constructed UPGMA tree by solely IGS1 sequences separated the samples into two main groups of species which was not desirably able to classify the species (Figure 2). However, the construction of a phylogenetic tree based on integration of IGS1 and ITS sequences resulted in a confident taxonomic conclusion. In total, the bootstrap values obtained in IGS1 tree were lower than the combination of two regions (Figures 2 and 4). At the first clade of both trees, *P. pulmonarius* members presented high homology with the previously deposited sequences of *P. pulmonarius* (AB234031 and AB115050 from Japan) [23]. The phylogenetically different species of *P. floridanus* (FPFMK), *P. citrinopileatus* (FPCMC), and *P. sapidus* (FPSPT) that were wrongly categorized in a single clade by IGS1 sequences could clearly be distinguished by integration of IGS1 and ITS sequences. Another distinct achievement of combined IGS1 and ITS regions compared to solely IGS1 was the provision of more precise classification and discrimination of *P. eryngii*, *P. eryngii *var. *ferulae*, and *P. nebrodensis* (Figure 4). Other evaluated species and strains were clearly distinguished by the combined IGS1 and ITS phylogenetic tree. Moreover, the experimental species and strains were confirmed by additional material which were downloaded from the NCBI GenBank and supported by high bootstrap values.

**Figure 3:**
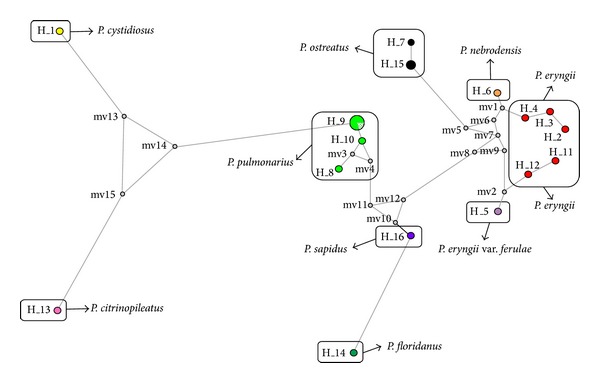
The median joining haplotype network of the combined IGS1 and ITS sequences. H and MV indicate haplotype and median vector, respectively.

**Figure 4:**
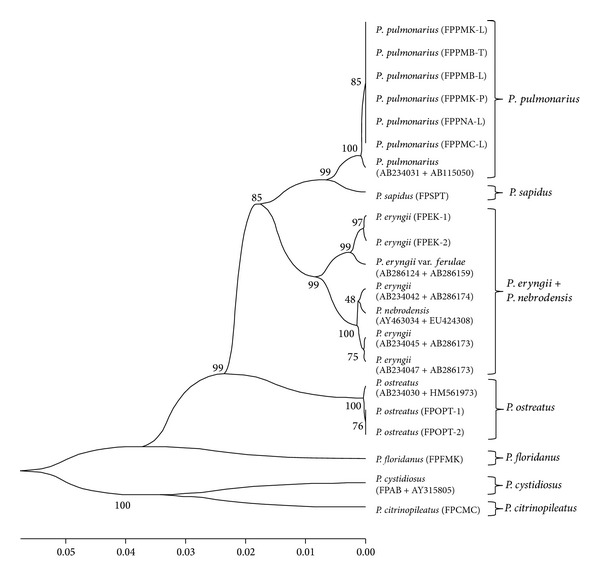
Phylogenetic tree constructed by the combination of IGS1 and ITS sequences using UPGMA method. The IGS1 sequences of AB234031 (*P. pulmonarius*); AB234030 (*P. ostreatus*); AB234045, AB234042, and AB234047 (*P. eryngii*); AY463034 (*P. nebrodensis*); and AB286124 (*P. eryngii *var. *ferulae*) as well as ITS sequences of HM561973 (*P. ostreatus*); AB115050 (*P. pulmonarius*); AB286159 (*P. eryngii *var. *ferulae*); AB286173 and AB286174 (*P. eryngii*); EU424308 (*P. nebrodensis*); and AY315805 (*P. cystidiosus*) were downloaded from NCBI GenBank. Numbers close to branches indicate 1000 replications of bootstrap test and codes represent the sample ID.

The relationships between combined IGS1 and ITS haplotypes were profoundly assessed by constructing the genealogical medianjoining network (Figure 3). Results obtained showed that 16 haplotypes belonged to eight species and one variety could clearly be distinguished. According to the network tree, haplotype 1 (H_1 : *P. cystidiosus*) and H_13 (*P. citrinopileatus*) were segregated from the other species by a number of mutations. H_1, H_13, and H_9 (*P. pulmonarius*) were connected together by 3 median vectors (13, 14, and 15). Median vectors (MV) obtained in this study can demonstrate an extinct ancestral strain or possible extant unsampled sequences [37, 38]. A distinct outcome that made MJ network analysis superior than phylogenetic tree emerged when a greater discrimination of *P. pulmonarius* members was demonstrated. H_8 (*P. pulmonarius*, AB234031 + AB115050) was connected to H_10 (*P. pulmonarius*, Thai strain) by mv3, and H_10 consequently linked to H_9 (*P. pulmonarius*, local strains) by a mutation. However, PL27 which is one of the available local strains of *P. pulmonarius* in Malaysia could not be distinguished and located in H_9 (indicated by a parallel hatched pattern). This can prove that PL27 may be an inbreed hybrid of local strain or originated from the same location. The positions of *P. sapidus*, *P. floridanus*, and *P. ostreatus* were clearly illustrated. Other distinct evidence that made network analysis more effective than phylogenetic tree appeared where H_5 (*P. eryngii* var. *ferulae*) and H_6 (*P. nebrodensis*) could be discriminated by mv2 and mv1, respectively (Figure 3). However, according to the combined IGS1 and ITS phylogenetic tree, the position of these species was not clearly observed (Figure 4).

## 4. Discussion

The identification of precise taxonomic position of species is essential when selection programs are being employed for any new strain developmental experiments as well as for any newly designed mushroom breeding programs. As for domestication and hybridization breeding, the intra- and interspecies information is highly valuable. ITS region will be a useful marker for interspecies information that allows farmers to recognize the species of interest for crossbreeding, hybrid vigour, and minimizing of inbreeding effects. Farmers can then have records of movement of species within and between farms. This will give farmers an opportunity not to arrive at an erroneous conclusion by recognizing the targeted species as an incorrect species [39, 40]. As noted earlier, a number of molecular based markers have been hitherto employed to investigate phylogenetic relationship and taxonomic hierarchy of the edible mushrooms, particularly *Pleurotus* genus [9, 41]. However, many such studies on fungal strain identification have been performed by common morphological characteristics [5, 11] which were unreliable and misleading [42, 43].

The current study revealed that *P. pulmonarius* was placed in a distinct clade with the *P. pulmonarius* strains referenced from NCBI GenBank (AB234031 + AB115050 from Japan) [23]. Based on the study done by Fries [44], this species nominated as *L. sajor-caju* was later renamed as *P. sajor-caju* by Singer [45]. However, based on morphological traits and microscopic comparisons done by Pegler and Yi-Jian [46], it was proposed that *L. sajor-caju* should not be categorized as *P. sajor-caju*. Another study done by Li and Yao [47] based on molecular and morphological data led to the findings and agreement that the scientific name of this species is *P. pulmonarius*. However, the most recent report done by Shnyreva et al. [48] confirmed that *P. sajor-caju* and *P. pulmonarius* are two separate species. The present study revealed that the available reference sequences identified as *P. pulmonarius* in the NCBI database, in accordance with taxonomic classification, suggested that the strains used in this study may relate to *P. pulmonarius.*

The phylogenetic tree based on integration of IGS1 and ITS regions assisted towards the proper classification of species and strains (Figure 4). However, the solely implemented IGS1 sequences resulted in a tree which compiled three distinct species of *P. floridanus, P. citrinopileatus*, and *P. sapidus* in one clade (Figure 2). Moreover, the IGS1 tree was not able to distinguish species of *P. nebrodensis *and *P. eryngii* var. *ferulae* from *P. eryngii*. In addition to the ambiguities generated by using single IGS1, prior studies on other molecular markers have also pointed out various ambiguities. Great similarity was reported between *P. pulmonarius *and *P. eryngii* (96% bootstrap support) as well as between *P. sapidus* and *P. colombinus* (100% bootstrap support) by Gonzalez and Labarère [11]. However, Zervakis et al. [49] and Iracabal et al. [50] disclosed a close relationship between *P. ostreatus* and *P. colombinus* and    *P. eryngii *and *P. cystidiosus*. In total, the positions of *P. pulmonarius*, *P. sapidus*, *P. sajor-caju*, *P. colombinus*, *P. eryngii*, *P. flabellatus*, and *P. cystidiosus* have remained ambiguous and some of these species have been wrongly grouped together in the same branch [11]. However, the acquired phylogenetic tree based on combined sequences of IGS1 and ITS regions provided such variation within and among species that could clearly delimit the examined species and strains.

In the present study, desirable AMOVA results were achieved when IGS1 and ITS sequences of the examined samples were combined. The combined regions gave better genetic differentiation among and within species with a high genetic variation of 91.82% and 8.18%, respectively (Table 4).

Though the parsimony network analysis can deliver a better understanding of the relationships within the genus, based on the precise literature, no record was found to show the use of this analysis for *Pleurotus* genus. In spite of achieving a proper classification of species by integration of IGS1 and ITS sequences, the phylogenetic tree showed the misclassification of the species of *P. nebrodensis* and *P. eryngii* var. *ferulae* with other strains of *P. eryngii*. In contrast, the median joining network could differentiate between the above-mentioned species (Figure 3). Moreover, *P. pulmonarius* (H_10) which was previously grouped together with *P. pulmonarius* (H_9) by phylogenetic tree proved to be distinguishable by MJ network analysis. Hence, by employment of the MJ network tree as well as combination of IGS1 and ITS regions, the experimental species and strains used in the present study were properly discriminated.

According to Meyer and Paulay [51], two principal elements are proposed in DNA barcoding: (1) the ability to assign an unknown sample to a known species and (2) the ability to detect previously unsampled species as distinct. This study proved that the combination of ITS and IGS regions with demonstrating simple amplification, location between conserved ribosomal genes, and adequate divergence levels (among and within populations) can be employed as a fungal DNA barcode. Moreover, according to Gilmore et al. [52], Nguyen and Seifert [12], and Vialle et al. [53], DNA barcoding and sequence-based molecular identification are known to be a small and standardized DNA fragment which could be amplified simply. However, the amplification and combination of IGS1 and ITS regions in this study required extra work and also the obtained fragments were not small and standard (>1.1 kb). Furthermore, the prospect of assigning an unknown to a known is promising especially for well-known, comprehensively sampled groups [51]. Moreover, according to Dupuis et al. [25], it has been shown that all marker groups have relatively equal success in delineating closely related species and that using more markers increases average delimitation success. Thus, in order to discover a consensus and powerful DNA barcodes for identification of fungal strains and species, this study suggests that more comprehensive sampled groups should be employed and/or new regions and molecular markers should be developed and consequently a comparison study should be conducted.

In conclusion, this study demonstrated the potential of the integration of IGS1 and ITS sequences of common cultivated *Pleurotus* species as an efficient inter- and intraspecies DNA marker. This marker could be used as a sequence-based DNA barcode because it presented sufficient variation among and within species, distinct sequences, simple amplification, and location between ideal conserved ribosomal genes. However, this study suggests that new molecular markers should be developed which can easily be employed for identification of the *Pleurotus* genus. Moreover, due to nonavailability of comprehensive sampled groups, we suggest examination of this highly variable marker with a greater sample size. The analyses used in the current study such as the medianjoining network, phylogenetic trees, AMOVA, haplotype data file, and sites information could provide a wide understanding of the species' relationships. To sum up, integration of IGS1 and ITS regions is recommended for identification of mushroom species in breeding programs including the domestication of native edible fungi, studies on structure mating types, and the production of high yield hybrids.

## Figures and Tables

**Table 1:** Strains and species used in this study and the GenBank accession numbers.

Strain(s)	Species	Location	Product length (bp)		GenBank (NCBI) accession number	
			IGS1	ITS1&2	IGS1	ITS1&2
FPPMK-L	*Pleurotus pulmonarius* Local strain (Reishi Lab Sdn. Bhd.)	Malaysia	530	660	JX271874	JX429930
FPPMB-L	*Pleurotus pulmonarius* Local strain (Damansara Mushroom Industry)	Malaysia	530	660	JX271878	JX429931
FPPNA-L	*Pleurotus pulmonarius* Local strain (NAS Agro Farm)	Malaysia	530	660	JX271879	JX429932
FPPMC-L	*Pleurotus pulmonarius* Local strain (GanoFarm Sdn. Bhd.)	Malaysia	530	660	JX271880	JX429933
FPPMK-P	*Pleurotus pulmonarius* PL27 (Reishi Lab Sdn. Bhd.)	Malaysia	530	660	JX271881	JX429934
FPPMB-T	*Pleurotus pulmonarius* Thai strain (Damansara Mushroom Industry)	Malaysia	531	660	JX271889	JX429935
FPCMC	*Pleurotus citrinopileatus* (GanoFarm Sdn. Bhd.)	Malaysia	527	666	JX271882	JX429936
FPFMK	*Pleurotus floridanus* (Reishi Lab Sdn. Bhd.)	Malaysia	527	668	JX271884	JX429937
FPOPT-1	*Pleurotus ostreatus* Gift from Mycobiotech	Singapore	543	669	JX271885	JX429938
FPOPT-2	*Pleurotus ostreatus* Gift from Mycobiotech	Singapore	543	669	JX271886	JX429939
FPSPT	*Pleurotus sapidus* Gift from Institute of Edible Mushroom (Shanghai, China)	China	527	668	JX271888	JX429940
FPAB	*Pleurotus cystidiosus* (GanoFarm Sdn. Bhd.)	Malaysia	530	—	JX271891	—
FPEK-1	*Pleurotus eryngii* (Obtained from supermarket, imported from Thailand)	Thailand	548	668	JX271883	JX429941
FPEK-2	*Pleurotus eryngii* (Obtained from supermarket, imported from China)	China	548	668	JX271887	JX429942

**Table 2:** List of designed and referenced PCR primers used in this study.

Primer ID	Type	Sequence	*T*_*m*_	GC%
LR12R	Forward	5′-CTGAACGCCTCTAAGTCAGAA-3′	53°C	—
LR13R	Forward	5′-GCATTGTTGTTCCGATG-3′	53°C	—
5SRNA	Reverse	5′-ATCAGACGGGATGCGGT-3′	53°C	—
ITS1	Forward	5′-TCCGTAGGTGAACCTGCGG-3′	55°C	—
ITS1f	Forward	5′-TCCTCCGCTTATTGATATGC-3′	57°C	—
ITS4	Reverse	5′-CTTGGTCATTTAGAGGAAGTAA-3′	55°C	—
ITS4B	Reverse	5′-CAGGAGACTTGTACACGGTCCAG-3′	57°C	—
IGS1-UM4	Forward	5′-AGTAAACTGACTTCAATTTCCGAGC-3′	55°C	40
IGS1-UM5	Reverse	5′-ATCCGCTGAGGTTAAGCCCT-3′	55°C	55
ITS1-UM2	Forward	5′-TAACAAGGTTTCCGTAGGTG-3′	55°C	45
ITS2-UM2	Reverse	5′-CTTAAGTTCAGCGGGTAGTC-3′	55°C	50

**Table 3:** The reference sequences used in this study, downloaded from NCBI GenBank.

IGS1			ITS1&2		
GenBank ID	Species	Location	GenBank ID	Species	Location
AB234042	*P. eryngii *	Japan	AB286173	*P. eryngii *	Japan
AB234045	*P. eryngii *	Japan	AB286174	*P. eryngii *	Japan
AB234047	*P. eryngii *	Japan	AY315805	*P. cystidiosus *	Japan
AB286142	*P. eryngii *	Japan	HM561973	*P. ostreatus *	Singapore
AB286124	*P. eryngii *var. *ferulae *	Japan	AB286159	*P. eryngii *var. *ferulae *	Japan
AY463034	*P. nebrodensis *	China	EU424308	*P. nebrodensis *	China
AB234031	*P. pulmonarius *	Japan	AB115050	*P. pulmonarius *	Japan
AB234030	*P. ostreatus *	Japan	—	—	—

**Table 4:** Proposed features of IGS1, ITS, and the combination of IGS1 and ITS regions computed in the current study.

Region	Number of sequences considered	Variation among population (%)	Variation within population (%)	Conserved sites	Variable sites	Parsimony informative sites	Singleton sites	Total number of haplotypes	Overall mean distance (diversity)
IGS1	22	88.98	11.02	512/555 92.25%	42/555 7.57%	30/555 5.41%	12/555 2.16%	14	0.019
ITS1&2	19	92.73	7.27	447/704 63.49%	239/704 33.95%	105/704 14.91%	124/704 17.61%	12	0.043
IGS1 + ITS1&2	21	91.82	8.18	958/1259 76.09%	282/1259 22.40%	141/1259 11.20%	131/1259 10.41%	16	0.051
